# Synthesis of C2-Alkoxy-Substituted 19-Nor Vitamin D_3_ Derivatives: Stereoselectivity and Biological Activity

**DOI:** 10.3390/biom12010069

**Published:** 2022-01-04

**Authors:** Yuka Mizumoto, Ryota Sakamoto, Akiko Nagata, Suzuka Sakane, Atsushi Kittaka, Minami Odagi, Masayuki Tera, Kazuo Nagasawa

**Affiliations:** 1 Department of Biotechnology and Life Science, Faculty of Engineering, Tokyo University of Agriculture and Technology, 2-24-16, Naka-cho, Koganei, Tokyo 184-8588, Japan; s200343y@st.go.tuat.ac.jp (Y.M.); s218969x@st.go.tuat.ac.jp (R.S.); s193819x@st.go.tuat.ac.jp (A.N.); ss4ever1124@gmail.com (S.S.); odagi@cc.tuat.ac.jp (M.O.); tera@go.tuat.ac.jp (M.T.); 2 Faculty of Pharmaceutical Science, Teikyo University, 2-11-1, Kaga, Itabashi-ku, Tokyo 173-8605, Japan; akittaka@pharm.teikyo-u.ac.jp

**Keywords:** C2-substitution, 19-norvitamin D_3_, Julia olefination, stereoselectivity, VDR binding affinity, cell differentiation

## Abstract

The active form of vitamin D_3_ (D_3_), 1a,25-dihydroxyvitamn D_3_ (1,25D_3_), plays a central role in calcium and bone metabolism. Many structure–activity relationship (SAR) studies of D_3_ have been conducted, with the aim of separating the biological activities of 1,25D_3_ or reducing its side effects, such as hypercalcemia, and SAR studies have shown that the hypercalcemic activity of C2-substituted derivatives and 19-nor type derivatives is significantly suppressed. In the present paper, we describe the synthesis of 19-nor type 1,25D_3_ derivatives with alkoxy groups at C2, by means of the Julia–Kocienski type coupling reaction between a C2 symmetrical A ring ketone and a CD ring synthon. The effect of C2 substituents on the stereoselectivity of the coupling reaction was evaluated. The biological activities of the synthesized derivatives were evaluated in an HL-60 cell-based assay. The a-methoxy-substituted **C2α-7a** was found to show potent cell-differentiating activity, with an ED_50_ value of 0.38 nM, being 26-fold more potent than 1,25D_3_.

## 1. Introduction

**Figure 1:**
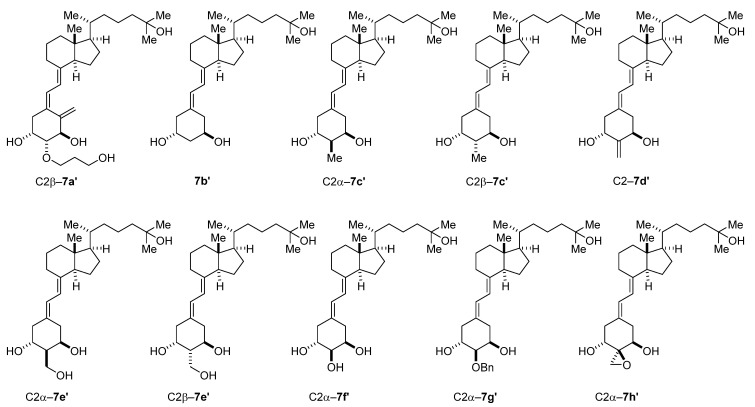
Structures of 19-nor type vitamin D derivatives substituted at C2.

The active form of vitamin D_3_ (D_3_), 1a,25-dihydroxyvitamin D_3_ (1,25D_3_), is involved in various physiological activities, including calcium metabolism, cell differentiation, and immunomodulation, via binding to the vitamin D receptor (VDR) [1,2,3,4]. Various derivatives of 1,25D_3_ have been synthesized and their structure–activity relationships (SARs) have been investigated, with the aim of separating the diverse biological activities of 1,25D_3_ or reducing side effects, such as hypercalcemia (Figure 1). It has been found that substituents at C2 have significant effects on the binding affinity to VDR, as well as on calcium metabolism and cell-differentiating activity. For example, a derivative of ED-71 (**C2β-7a’**) bearing a hydropropoxy group at C2, shows bone formation activity comparable to that of 1,25D_3_ despite its weak binding affinity for VDR [5,6]. Furthermore, 19-nor type derivatives (**7b’**) have been reported to show suppressed hypercalcemic activity, while retaining cell differentiation-inducing activity [7]. Thus, there is considerable interest in the synthesis and biological activities of 19-nor type derivatives bearing a substituent at C2.

Many C2-substituted 19-nor type derivatives with C–C bonds or hydroxyl groups have already been synthesized. In 1998, Sicinski et al. reported a synthesis of 19-nor type derivatives with C2 substituents, such as methyl (**C2-7c’**), methylene (**C2-7d’**), and hydroxymethyl groups (**C2-7e’**) linked through C–C bonds, and found that the α-methyl-substituted derivative shows strong VDR-binding and HL-60 cell differentiation-inducing activities [8]. The same group also synthesized 19-nor type vitamin D_3_ with a 2α-hydroxyl group at C2 (**C2α-7f’**), and reported that its VDR-binding affinity was about 1/5th of that of 1,25D_3_, while the HL-60 cell differentiation-inducing activity was comparable to that of 1,25D_3_ [9,10]. In addition to the above examples, many other derivatives have been synthesized, but few 19-nor type derivatives with alkoxy substituents at C2 have been reported, and their biological activities have been little investigated [11,12,13,14]. A series of C2-alkoxy-substituted 19-nor type derivatives with α-benzyloxy or epoxy groups has been synthesized and evaluated. In the case of the α-benzyloxy-substituted derivative (**C2α-7g’**), the HL-60 cell differentiation activity was decreased to ca 1/10th of that of 1,25D_3_ [10]. The 2α-epoxy-substituted derivative (**C2α-7h’**) showed a low VDR binding affinity, only1/25th of that of 1,25D_3_, and the affinity of the 2β-derivative was even lower [15].

In this study, we synthesized 19-nor D_3_ derivatives with a methoxy, benzyloxy, or p-nitrophenoxy group at C2 by means of Julia-type coupling, between a C2-symmetric A ring ketone and a CD ring synthon. In the coupling reaction, we observed interesting effects of substituents at C2, on the diastereoselectivity of the coupling reaction, and these effects are discussed in terms of the transition state of the coupling reaction [16]. The VDR binding affinity and HL-60 cell differentiation-inducing activity were evaluated. Among these compounds, the 2α-methoxy-substituted derivative showed ca 26-fold more potent cell differentiation-inducing activity than 1,25D_3_, while its affinity for VDR was similar to that of 1,25D_3_.

## 2. Materials and Methods

### 2.1. Chemistry

Flash chromatography was performed using silica gel 60 (spherical, particle size 0.040–0.100 mm. Kanto Co., Inc., Tokyo, Japan), and preparative-TLC (PLC) was performed using PLC Silica gel 60 F254 (0.5 mm, Merck Ltd., Darmstadt, Germany). Optical rotations were measured on a JASCO P-2200 polarimeter. The ^1^H and ^13^C NMR spectra were recorded on JEOL JNM-AL300 (300 MHz), JEOL JNM-ECX 400 (400 MHz) and JEOL JNM-ECA 500 (500 MHz). Chemical shift in chloroform-d was reported in the scale relative to chloroform-*d* (7.26 ppm) for ^1^H NMR, respectively. For ^13^C NMR, chemical shift was reported in the scale relative to chloroform-d (77.0 ppm) as an internal reference. Mass spectra were recorded on JEOL JMS-T100LC spectrometer. HPLC analysis was performed on Elite LaChrom HPLC (Hitachi, Ltd., Tokyo, Japan), Senshu pak PEGASIL Silica 60–5 column and SHISEIDO CAPCELPAK (ADME, Osaka, Japan) column, with *n*-hexane/2- propanol and acetonitrile/water as eluent.

(7*R*,9*R*)-7,9-bis((tert-butyldimethylsilyl)oxy)-2-(4-methoxyphenyl)-1,3-dioxaspiro[4.5]decan-8-ol (**3**). Both *p*-anisaldehyde dimethyl acetal (255 μL, 1.50 mmol) and (+)-10-Camphorsulfonic Acid (18.8 mg, 0.075 mmol) at 0 °C were added to a solution of (3R,5R)-3,5-bis((tert-butyldimethylsilyl)oxy)-1-(hydroxymethyl)cyclohexane-1,4-diol **2** [17] (610 mg, 1.50 mmol) in dry CH_2_Cl_2_ (3 mL). The reaction mixture was allowed to warm to room temperature. After being stirred for 2.5 h, the mixture was poured into saturated aqueous NaHCO_3_ and extracted with CH_2_Cl_2_. The combined organic layer was washed with brine, dried over MgSO_4_ and concentrated in vacuo. The residue was purified by column chromatography on silica gel (*n*-hexane/ethyl acetate = 50:1) afforded **3** (773 mg, 98%) as a mixture of isomers. ${\left[a\right]}_{D}^{25}$ = −4.2 (*c* 17.31, CHCl_3_); ^1^H NMR (300 MHz, CDCl_3_, 1:1 mixture of isomers) *δ* 7.41 (dd, *J* = 8.3, 6.5 Hz, 4H), 6.90 (dd, *J* = 8.6, 2.1 Hz, 4H), 5.88 (s, 1H), 5.76 (s, 1H), 4.16 (t, *J* = 3.4 Hz, 2H), 4.06–3.93 (m, 4H), 3.88–3.80 (m, 8H), 3.61 (t, *J* = 3.1 Hz, 2H), 2.47 (s, 2H), 2.25 (ddd, *J* = 13.7, 4.7, 3.0 Hz, 2H), 2.11–1.85 (m, 4H), 1.80–1.68 (m, 2H), 0.92–0.90 (m, 36H), 0.12–0.08 (m, 24H); ^13^C NMR (75 MHz, CDCl_3_, mixture of isomers) *δ* 160.27, 160.17, 130.64, 129.94, 127.98, 127.75, 113.65, 102.27, 101.15, 80.52, 79.89, 76.11, 74.84, 72.30, 72.13, 69.76, 69.56, 68.30, 67.66, 55.18, 39.25, 37.07, 36.42, 36.22, 25.74, 25.69, 18.01, 17.82, −4.70, −4.92, −5.05, −5.23; HRMS (ESI, M + Na) calcd. for 547.28871, found 547.28650.

(((7*R*,9*R*)-8-methoxy-2-(4-methoxyphenyl)-1,3-dioxaspiro[4.5]decane-7,9-diyl)bis(oxy))bis(tert-butyldimethylsilane) (**4a**). NaH (60% dispersion in mineral oil, 61.0 mg, 1.52 mmol) at 0 °C was added to a solution of **3** (200 mg, 0.381 mmol) in dry THF (7.6 mL). After being stirred for 30 min, iodomethane (119 μL, 1.91 mmol) was added dropwise to the mixture. The reaction mixture was allowed to warm to room temperature. After stirring for 12 h, the mixture was poured into water and extracted with CH_2_Cl_2_. The combined organic layer was washed with brine, dried over MgSO_4_ and concentrated in vacuo. The residue was purified by column chromatography on silica gel (*n*-hexane/ethyl acetate = 70:1) to yield a crude product.

(((7*R*,9*R*)-8-(benzyloxy)-2-(4-methoxyphenyl)-1,3-dioxaspiro[4.5]decane-7,9-diyl)bis(oxy))bis(tert-butyldimethylsilane) (**4b**). NaH (60% dispersion in mineral oil, 152.5 mg, 3.81 mmol) at 0 °C was added to a solution of **3** (500 mg, 0.953 mmol) in dry THF (19.1 mL) [18]. After being stirred for 30 min, (Iodomethyl)benzene [19] (593 μL, 4.76 mmol) was added dropwise to the mixture. The reaction mixture was allowed to warm to room temperature. After stirring for 12 h, the mixture was poured into water and extracted with CH_2_Cl_2_. The combined organic layer was washed with brine, dried over MgSO_4_ and concentrated in vacuo. The residue was purified by column chromatography on silica gel (*n*-hexane/ethyl acetate = 70:1) to yield a crude product.

(((7*R*,9*R*)-2-(4-methoxyphenyl)-8-(4-nitrophenoxy)-1,3-dioxaspiro[4.5]decane-7,9-diyl)bis(oxy))bis(tert-butyldimethylsilane) (**4c**). NaH (60% dispersion in mineral oil, 57.2 mg, 1.43 mmol) at room temperature was added to a solution of **3** (150 mg, 0.286 mmol) in dry DMF (9.5 mL) [18]. After being stirred for 10 min, 4-fluoro nitrobenzene (91.0 μL, 0.857 mmol) was added dropwise to the mixture. After stirring for 30 min, the mixture was poured into water and extracted with AcOEt. The combined organic layer was washed with brine, dried over MgSO_4_ and concentrated in vacuo. The residue was purified by column chromatography on silica gel (*n*-hexane/ethyl acetate = 40:1) to yield a crude product.

General procedure for the synthesis of (3*R*,5*R*)-3,5-bis((tert-butyldimethylsilyl)oxy)-4-*R*-cyclohexan-1-one (**5**). Pyridinium *p*-toluenesulfonate (15 eq) at room temperature was added to a solution of the crude product **4** in MeOH (0.012 M). After being stirred for 15 min, the mixture was poured into saturated aqueous NaHCO_3_ and extracted with AcOEt. The combined organic layer was washed with brine, dried over MgSO_4_ and concentrated in vacuo. The residue was purified by column chromatography on silica gel (*n*-hexane/ethyl acetate = 10:1) to yield a crude product.

Sodium periodate (6 eq) at room temperature was added to a solution of the crude product in MeOH/distilled water (7:1, 0.02 M). After being stirred for 2 h, the mixture was poured into water and extracted with AcOEt. The combined organic layer was washed with brine, dried over MgSO_4_ and concentrated in vacuo. The residue was purified by column chromatography on silica gel (*n*-hexane/ethyl acetate = 20:1) and afforded **5** as a colorless oil.

(3*R*,5*R*)-3,5-bis((tert-butyldimethylsilyl)oxy)-4-methoxycyclohexan-1-one (**5a**). Yield 33%, 3 steps. ${\left[a\right]}_{D}^{25}$ = −6.9 (*c* 5.67, CHCl_3_); ^1^H NMR (300 MHz, CDCl_3_) *δ* 4.32 (qd, *J* = 4.8, 2.3 Hz, 1H), 4.19 (q, *J* = 4.6 Hz, 1H), 3.57 (s, 3H), 3.35 (q, *J* = 2.5 Hz, 1H), 2.73–2.63 (m, 2H), 2.44–2.38 (m, 1H), 2.25–2.16 (m, 1H), 0.88 (s, 6H), 0.84 (d, *J* = 2.8 Hz, 6H), 0.06 (t, *J* = 2.6 Hz, 12H); ^13^C NMR (75 MHz, CDCl_3_) *δ* 207.90, 82.53, 76.57, 68.90, 59.71, 46.69, 45.25, 25.72, 25.58, 18.04, 17.85, −4.90, −4.99; HRMS (ESI, M + Na) calcd. for 411.23628, found 411.23427.

(3*R*,5*R*)-4-(benzyloxy)-3,5-bis((tert-butyldimethylsilyl)oxy)cyclohexan-1-one (**5b**). Yield 60%, 3 steps. ${\left[a\right]}_{D}^{25}$ = −16.4 (*c* 9.47, CHCl_3_); ^1^H NMR (300 MHz, CDCl_3_) *δ* 7.37–7.26 (m, 5H), 4.99 (d, *J* = 12.0 Hz, 1H), 4.69 (d, *J* = 11.7 Hz, 1H), 4.35 (dq, *J* = 10.6, 2.4 Hz, 1H), 4.13 (q, *J* = 4.2 Hz, 1H), 3.62 (d, *J* = 2.4 Hz, 1H), 2.86–2.71 (m, 2H), 2.44 (dd, *J* = 14.1, 4.5 Hz, 1H), 2.20 (dd, *J* = 15.3, 2.6 Hz, 1H), 0.91 (d, *J* = 3.1 Hz, 9H), 0.85–0.81 (m, 9H), 0.08 (d, *J* = 4.5 Hz, 6H), −0.01 (dd, *J* = 8.9, 5.8 Hz, 6H); ^13^C NMR (75 MHz, CDCl_3_) *δ* 208.21, 138.72, 128.37, 127.67, 79.48, 73.79, 69.35, 46.78, 44.95, 25.79, 25.56, 18.07, 17.80, −4.82, −5.14; HRMS (ESI, M + Na) calcd. for 487.26758, found 487.26393.

(3*R*,5*R*)-3,5-bis((tert-butyldimethylsilyl)oxy)-4-(4-nitrophenoxy)cyclohexan-1-one (**5c**). Yield 20%, 3 steps. ${\left[a\right]}_{D}^{25}$ = −27.1 (*c* 0.87, CHCl_3_); ^1^H NMR (400 MHz, CDCl_3_) *δ* 8.21–8.18 (m, 2H), 6.94 (td, *J* = 6.3, 3.8 Hz, 2H), 4.83 (q, *J* = 5.5 Hz, 1H), 4.25 (qd, *J* = 4.0, 2.1 Hz, 1H), 4.14 (dd, *J* = 6.2, 2.1 Hz, 1H), 2.91 (ddd, *J* = 15.3, 4.8, 1.4 Hz, 1H), 2.77–2.71 (m, 1H), 2.56–2.49 (m, 2H), 0.90–0.88 (m, 18H), 0.15 (t, *J* = 3.0 Hz, 3H), 0.11 (s, 3H), 0.09–0.06 (m, 6H); ^13^C NMR (100 MHz, CDCl_3_) *δ* 205.44, 162.12, 141.92, 126.03, 115.29, 75.79, 72.86, 69.93, 46.66, 42.17, 25.85, 25.79, 18.17, 18.12, −4.24, −4.56, −4.77; HRMS (ESI, M + Na) calcd. for 518.23701, found 518.23458.

General procedure for the synthesis of (1*R*,3*R*)-5-(2-((1*R*,3a*S*,7a*R*,*E*)-1-((*S*)-6-hydroxy-6-methylheptan-2-yl)-7a-methyloctahydro-4H-inden-4-ylidene)ethylidene)-2-alkoxy-1,3-diol (**7**). LiHMDS (1.3 M solution in THF, 41 μL) at −78 °C was added to a solution of CD rings **6** (24.0 mg, 0.045 mmol) in dry THF (0.4 mL). After the mixture was stirred at the same temperature for 1 h, a solution of A ring 5 (0.041 mmol) in dry THF (0.4 mL) was added dropwise to the mixture. After being stirred for 1 h, the mixture was poured into saturated aqueous NH_4_Cl and extracted with AcOEt. The combined organic layer was washed with brine, dried over MgSO_4_ and concentrated in vacuo. The residue was purified by column chromatography on silica gel (*n*-hexane/ethyl acetate = 80:1) to yield crude protected 19-norvitamin D_3_. D_3_ in MeOH (0.04 M) and CH_2_Cl_2_ (0.04 M) was added (+)-CSA (6 eq) at room temperature to a solution of the crude protected 19-norvitamin D. After being stirred at rt for 12 h, the reaction mixture was diluted with AcOEt. The resulting mixture was washed with brine, dried over MgSO_4_ and concentrated. Purification by silica gel column chromatography (*n*-hexane/ethyl acetate = 1:1) afforded **7** as a mixture of isomers. The isomers were separated by reversed-phase HPLC (Senshu pak PEGASIL Silica 60–5 column, 5 mm, 250 mm × 10 mm, 2.0 mL/min) using *n*-hexane/2- propanol (55:45) for C2α-**7a** and C2β-**7a**; and HPLC (SHISEIDO CAPCELPAK (ADME) column, 5 mm, 250 mm × 4.6 mm, 1.0 mL/min) using acetonitrile/water (70:30) for C2α-**7b** and C2β-**7b** [20]. Retention times: $t_{R}$ (C2α-**7a**) = 15.3 min; $t_{R}$ (C2β-**7a**) = 18.3 min;$\;t_{R}$ (C2α-**7b**) = 28.0 min; and$\;t_{R}$ (C2β-**7b**) = 29.1 min.

(1*R*,2*S*,3*R*,*E*)-5-(2-((1*R*,3a*S*,7a*R*,*E*)-1-((*S*)-6-hydroxy-6-methylheptan-2-yl)-7a-methyloctahydro-4H-inden-4-ylidene)ethylidene)-2-methoxycyclohexane-1,3-diol (C2α-**7a**). ${\left[a\right]}_{D}^{25}$ = 32.0 (*c* 0.15, CHCl_3_); ^1^H NMR (500 MHz, CDCl_3_) *δ* 6.34 (d, *J* = 10.9 Hz, 1H), 5.83 (d, *J* = 10.9 Hz, 1H), 4.15 (t, *J* = 2.6 Hz, 1H), 3.93 (td, *J* = 8.3, 4.4 Hz, 1H), 3.50 (s, 3H), 3.18 (q, *J* = 3.4 Hz, 1H), 2.80 (q, *J* = 6.7 Hz, 2H), 2.62 (dd, *J* = 13.5, 4.3 Hz, 1H), 2.25–2.14 (m, 2H), 2.00 (d, *J* = 12.0 Hz, 2H), 1.89–1.85 (m, 1H), 1.70–1.36 (m, 13H), 1.21 (s, 6H), 1.10–1.02 (m, 1H), 0.93 (d, *J* = 6.3 Hz, 3H), 0.55 (s, 3H); ^13^C NMR (125 MHz, CDCl_3_) *δ* 143.20, 130.00, 124.03, 115.41, 85.52, 71.11, 68.65, 66.20, 57.56, 56.50, 56.29, 45.75, 44.40, 41.12, 40.46, 36.37, 36.06, 32.62, 29.36, 29.17, 28.86, 27.64, 23.41, 22.27, 20.79, 18.79, 12.13; HRMS (ESI, M + Na) calcd. for 457.32938, found 457.32534.

(1*R*,2*R*,3*R*,*Z*)-5-(2-((1*R*,3a*S*,7a*R*,*E*)-1-((*S*)-6-hydroxy-6-methylheptan-2-yl)-7a-methyloctahydro-4H-inden-4-ylidene)ethylidene)-2-methoxycyclohexane-1,3-diol (C2β-**7a**). ${\left[a\right]}_{D}^{25}$ = 20.0 (*c* 0.26, CHCl_3_); ^1^H NMR (500 MHz, CDCl_3_) *δ* 6.29 (d, *J* = 11.4 Hz, 1H), 5.84 (d, *J* = 11.0 Hz, 1H), 4.22 (q, *J* = 3.2 Hz, 1H), 3.84–3.78 (m, 1H), 3.49 (s, 3H), 3.14–3.05 (m, 2H), 2.81–2.78 (m, 1H), 2.51–2.48 (m, 1H), 2.35 (d, *J* = 14.7 Hz, 1H), 1.64 (d, *J* = 40.8 Hz, 15H), 1.57–1.35 (m, 6H), 1.32–1.25 (m, 2H), 1.22 (s, 6H), 1.06 (q, *J* = 9.5 Hz, 1H), 0.93 (d, *J* = 6.4 Hz, 3H), 0.54 (s, 3H); ^13^C NMR (125 MHz, CDCl_3_) *δ* 143.13, 129.78, 123.80, 115.48, 86.48, 71.12, 68.79, 65.65, 57.24, 56.49, 56.29, 45.72, 44.40, 40.80, 40.45, 36.37, 36.08, 33.53, 29.34, 29.19, 28.99, 27.68, 23.52, 22.25, 20.79, 18.80, 12.05; HRMS (ESI, M + Na) calcd. for 457.32938, found 457.33158.

(1*R*,2*S*,3*R*,*E*)-2-(benzyloxy)-5-(2-((1*R*,3a*S*,7a*R*,*E*)-1-((*S*)-6-hydroxy-6-methylheptan-2-yl)-7a-methyloctahydro-4H-inden-4-ylidene)ethylidene)cyclohexane-1,3-diol (C2α-**7b**). ${\left[a\right]}_{D}^{25}$ = 32.6 (*c* 0.19, CHCl_3_); ^1^H NMR (500 MHz, CDCl_3_) *δ* 7.38–7.35 (m, 4H), 7.34 (dd, *J* = 7.7, 4.9 Hz, 1H), 6.33 (d, *J* = 11.5 Hz, 1H), 5.83 (d, *J* = 10.9 Hz, 1H), 4.74–4.62 (m, 2H), 4.11 (s, 1H), 3.97 (s, 1H), 3.45 (q, *J* = 3.4 Hz, 1H), 2.79 (dd, *J* = 14.3, 5.7 Hz, 2H), 2.65 (dd, *J* = 13.5, 4.3 Hz, 1H), 2.26 (d, *J* = 14.9 Hz, 1H), 2.18–2.13 (m, 2H), 2.08 (s, 1H), 2.00 (d, *J* = 12.0 Hz, 2H), 1.89–1.85 (m, 1H), 1.41–1.36 (m, 2H), 1.30–1.25 (m, 2H), 1.22 (d, *J* = 5.2 Hz, 8H), 1.11–1.03 (m, 1H), 0.93 (d, *J* = 6.9 Hz, 3H), 0.88–0.83 (m, 1H), 0.54 (d, *J* = 4.0 Hz, 3H); ^13^C NMR (125 MHz, CDCl_3_) *δ* 143.16, 137.92, 130.02, 128.68, 128.12, 127.84, 123.97, 115.44, 83.70, 72.05, 71.12, 68.82, 66.99, 56.51, 56.30, 45.77, 44.41, 41.08, 40.47, 36.38, 36.07, 32.68, 29.37, 29.18, 28.88, 27.65, 23.43, 22.27, 20.80, 18.80, 12.13; HRMS (ESI, M + Na) calcd. for 533.36068, found 533.35793.

(1*R*,2*R*,3*R*,*Z*)-2-(benzyloxy)-5-(2-((1*R*,3a*S*,7a*R*,*E*)-1-((*S*)-6-hydroxy-6-methylheptan-2-yl)-7a-methyloctahydro-4H-inden-4-ylidene)ethylidene)cyclohexane-1,3-diol (C2β-**7b**). ${\left[a\right]}_{D}^{25}$ = 13.8 (*c* 0.16, CHCl_3_); ^1^H NMR (500 MHz, CDCl_3_) *δ* 7.38–7.31 (m, 5H), 6.29 (d, *J* = 11.5 Hz, 1H), 5.84 (d, *J* = 10.9 Hz, 1H), 4.76–4.60 (m, 3H), 4.30 (q, *J* = 6.5 Hz, 1H), 4.18 (s, 1H), 3.86 (s, 1H), 3.37 (ddd, *J* = 15.6, 8.7, 3.0 Hz, 1H), 3.07 (dd, *J* = 13.5, 3.7 Hz, 1H), 2.81–2.78 (m, 1H), 2.49 (dd, *J* = 14.3, 2.9 Hz, 1H), 2.34 (d, *J* = 13.2 Hz, 1H), 2.27 (d, *J* = 2.3 Hz, 1H), 2.09–2.07 (m, 1H), 2.03–1.95 (m, 3H), 1.66 (t, *J* = 11.7 Hz, 2H), 1.41–1.36 (m, 2H), 1.32–1.25 (m, 4H), 1.21 (s, 8H), 1.06 (q, *J* = 9.7 Hz, 1H), 0.93 (d, *J* = 6.3 Hz, 4H), 0.54 (s, 3H); ^13^C NMR (125 MHz, CDCl_3_) *δ* 143.09, 137.78, 129.78, 128.71, 128.17, 127.93, 123.74, 115.49, 84.64, 71.73, 71.12, 68.85, 66.57, 56.48, 56.29, 45.72, 44.40, 40.81, 40.45, 36.37, 36.08, 33.51, 29.35, 29.19, 28.98, 27.67, 23.52, 22.26, 20.79, 18.80, 12.05; HRMS (ESI, M + Na) calcd. for 533.36068, found 533.36340.

(1*R*,2*S*,3*R*,*E*)-5-(2-((1*R*,3a*S*,7a*R*,*E*)-1-((*S*)-6-hydroxy-6-methylheptan-2-yl)-7a-methyloctahydro-4H-inden-4-ylidene)ethylidene)-2-(4-nitrophenoxy)cyclohexane-1,3-diol (C2α-**7c**). ${\left[a\right]}_{D}^{25}$ = 65.9 (*c* 0.54, CHCl_3_); ^1^H NMR (400 MHz, CDCl_3_) *δ* 8.22–8.19 (m, 2H), 7.03–7.00 (m, 2H), 6.34 (d, *J* = 11.0 Hz, 1H), 5.84 (d, *J* = 11.0 Hz, 1H), 4.63 (dt, *J* = 12.5, 4.8 Hz, 1H), 4.22 (s, 1H), 3.94 (dd, *J* = 7.8, 2.7 Hz, 1H), 2.93 (dd, *J* = 14.7, 5.0 Hz, 1H), 2.79–2.75 (m, 2H), 2.62 (s, 1H), 2.26 (dd, *J* = 21.8, 13.1 Hz, 2H), 2.02 (d, *J* = 11.9 Hz, 2H), 1.89 (d, *J* = 6.9 Hz, 1H), 1.22 (s, 6H), 0.93 (t, *J* = 6.9 Hz, 3H), 0.56 (s, 3H); ^13^C NMR (100 MHz, CDCl_3_) *δ* 162.85, 144.13, 141.68, 128.51, 126.00, 124.67, 115.58, 115.17, 77.75, 74.04, 71.14, 69.51, 56.49, 56.31, 45.85, 44.37, 40.42, 38.30, 36.36, 36.07, 32.24, 29.36, 29.17, 28.93, 27.63, 23.43, 22.26, 20.77, 18.79, 12.15; HRMS (ESI, M + Na) calcd. for 564.33011, found 564.33213.2.2. Biological Activity Evaluation.

Materials for biochemistry experiments: Vitamin D_3_ receptor (VDR), FluormoneTM VDR Red (Fluormone), VDR Red Assay Buffer and DTT solution were purchased from Invitrogen (PolarScreen Vitamin D Receptor Competitor Assay). Dimethyl sulfoxide (DMSO), 12-*O*-tetradecanoylphorbol-13 acetate (PMA) and nitroblue tetrazolium (NBT) were purchased from Sigma-Aldrich. RPMI 1640 media was purchased from Nissui Pharmaceutical, and fetal bovine serum (FBS) was purchased from Corning (collected in Brazil). A total of 384-well plates (polypropylene, flat bottom, 152 µL, black) were purchased from Greiner and 96-well plates from AGC Techno Glass. All other reagents were of molecular biology grade from Sigma-Aldrich, Wako Chemicals, or TCI.

Cell culture: Human promyelocytic leukemia cells (HL-60) were grown in RPMI supplemented with 10% heat-inactivated FBS in a CO_2_ incubator. The population doubling time of HL-60 cells was approximately 37 h under the conditions employed, and cells were passaged at 2.0 × 10^5^ cells/mL every 4 days. Cell viability was determined by trypan blue staining and was always more than 90%.

#### 2.1.1. Vitamin D Receptor Binding Analysis

The 1,25D_3_ and the synthesized derivatives were dissolved in DMSO (10 mM stock solutions) and diluted as required with DMSO and the VDR Red Assay Buffer. VDR and fluoromone in the VDR Red Assay Buffer solution (VDR 7.2 nM, fluoromone 2.0 nM, and 5.0 mM DTT, 15 µL) were added to the vitamin D_3_ derivatives (78 nM, DMSO 10%, 15 µL) in the VDR Red Assay Buffer solution and incubated for 2 h in a 384-well microplate at room temperature in the dark. Fluorescence polarization was measured on a SPARK-TKIS multimode plate reader (Tecan) using a 535 nm excitation filter (25 nm bandwidth) and a 590 nm emission filter (20 nm bandwidth). This experiment was performed three times for each vitamin D_3_ derivative. The VDR binding activity ratio was calculated as 9DmP_sample_/DmP_1,25D3_ × 100), where DmP = (mP without sample)–(mP with 39 nM vitamin D_3_ derivative or 1,25D_3_).

#### 2.1.2. Evaluation of the Differentiation-Inducing Activity by NBT Staining

HL-60 cells (2.0 × 10^5^ cells/mL) were cultured with or without vitamin D derivatives (1 × 10^−6^–10^−11^ M) in 200 µL of RPMI (0.1% DMSO) in a 96-well plate for 5 days in a CO_2_ incubator. Then, PMA (160 nM) and NBT (0.1%) were added and incubation was continued for 30 min in a CO_2_ incubator. The NBT-stained and non-stained cells were counted on a hemocytometer. The differentiation-inducing activity was calculated as (NBT-stained cell number)/(total cell number), in the presence of various concentrations of vitamin D derivatives or 1,25D_3_. EC_50_ values are given as the average of triplicate assays [21,22].

## 3. Results and Discussion

### 3.1. Chemistry

**Scheme 1:**
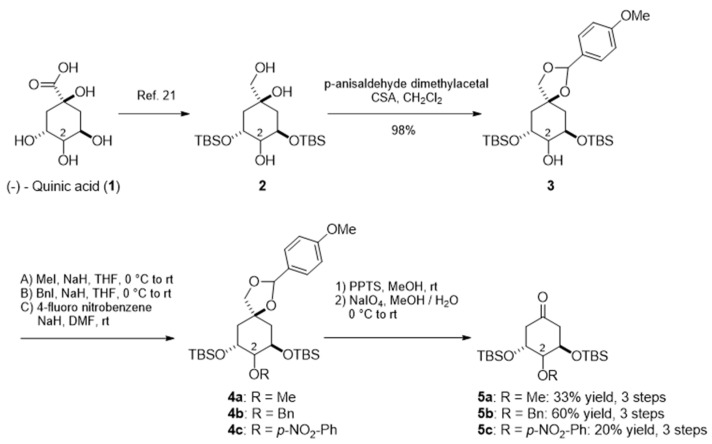
Synthesis of A ring ketones **5a**–**c**.

For the synthesis of the C2-alkoxy-substituted 19-nor type D_3_ derivatives, we firstly synthesized the A ring synthon ketones **5** bearing alkoxy-type substituents at C2, and examined the Julia–Lythgoe olefination reaction with the CD ring synthon **6**. Thus, the ketones **5a**–**c** bearing a methoxy, benzyloxy or 4-NO_2_-phenyloxy group were synthesized from diol **2** derived from (-)-quinic acid (**1**), as follows (Scheme 1) [23]. The diol **2** was reacted with *p*-anisaldehyde dimethyl acetal, using camphor sulfonic acid as a catalyst, to give benzylidene acetal **3**, and the resulting secondary alcohol was converted into a methoxy, benzyloxy or *p*-nitrophenoxy group by the reaction with methyl iodide, benzyl iodide, or 4-fluoro-*p*-nitrobenzene, respectively, to give ethers **4a**–**c**. the deprotection of the benzylidene acetal in **4** was conducted with PPTS, followed by oxidative cleavage of the resulting diol with sodium periodate to obtain A ring ketones **5a**–**c** in 20–60% yield from **4**.

The C2 alkoxy-substituted ketones **5a**–**c** were subjected to the Julia–Kocienski coupling reaction with the CD ring synthon, sulfone **6**, affording two diastereomers at C2. The selectivity was determined by ^1^H NMR [9,24] after the deprotection of the silyl ethers and MOM group at C25, and the results are summarized in Table 1. In the case of **5a**, with the methoxy group at C2, C2β-**7a** was obtained preferentially in a ratio of 1:3.5 (entry 1), while no selectivity was observed in the case of **5b** (entries 2). Interestingly, only one diastereomer, C2α-**7c**, was obtained in the case of **5c** with a *p*-NO_2_-phenyl ether group (entry 3) [25] [See Supplementary Materials].

**Scheme 2:**
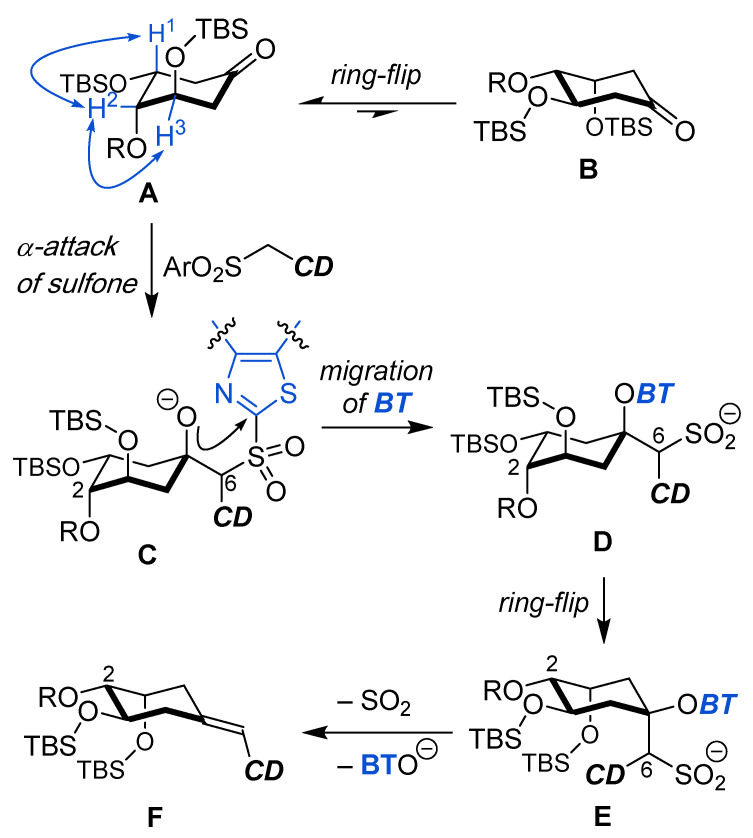
Conformational analysis of the A ring ketones **5** and mechanism of the Julia-type olefination.

These results suggest that the substituent at C2 influences the diastereoselectivity in the Julia-type coupling reaction, and therefore conformational analysis of the A ring ketones **5** bearing substituents at C2 was carried out by ^1^H NMR. Regardless of the substituent at C2, the ketones appear to preferentially take a particular chair conformation in the conformational equilibrium, as judged from the ^1^H NMR data (only small coupling constants of H^2^ were observed; *J*_H2–H1_ = 2.1–2.4 Hz, *J*_H2–H3_ = 2.1–2.5 Hz). Furthermore, the nuclear Overhauser effect (nOe) was observed between the H^2^ atom, and H^1^ and H^3^, respectively. Thus, the ketones adopted the chair conformation **A** with axial substituents at C1 and C2, as shown in Scheme 2. On the other hand, the A ring of the C2-substituted VD_3_ derivative is known to take a chair conformation, as shown in **F**, in which the C2 substituent occupies the equatorial position [9,24,26]. Thus, in the coupling reaction, the addition of sulfonate occurred from the a-face of the ketone to avoid steric hindrance with the C3 substituent, generating **C**. The BT (benzothiazole) group in the CD ring is transferred to the resulting hydroxyl group, and the ring conformation flips to **E** from **D**. Finally, the olefin is formed by the elimination of SO_2_ and BTO anion to generate **F**. Basically, in the Julia-type olefination reaction, two types of elimination pathways can occur, i.e., syn-periplanar elimination and anti-periplanar elimination, and the geometric isomerism of the generated olefin depends on these elimination processes.

**Figure 2:**
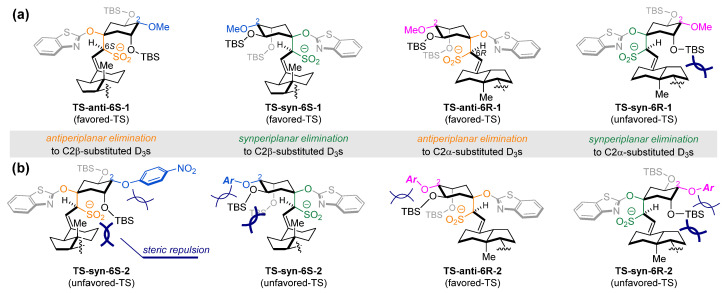
Possible transition states for the Julia-type olefination with **5a** (**a**) and **5c** (**b**).

The differences in the stereoselectivity of the coupling products of the Julia-type olefination with ketones **5,** can be attributed to the differences in the stability of the corresponding transition states (TS). There are four possible transition states resulting from differences in the mode of elimination (anti/syn) and the stereochemistry at the C6 position (*S*/*R*), and the TSs for **5a** with a methoxy group at C2 are shown in Figure 2a. Among them, **TS-syn-6R-1**, which leads to the C2α-product, would be unstable due to steric repulsion between the TBS group and the CD ring moiety. Therefore, the reaction would proceed through the three remaining states (**TS-anti-6S-1**, **TS-syn-6S-1** and **TS-anti-6R-1**), affording the C2β product preferentially. On the other hand, in the case of **5c** with a *p*-NO_2_-PhO group, this substituent is sterically hindered and would tend to push the TBS group towards the CD ring moiety, leading to steric repulsion between the TBS group and CD ring moiety, unfavorably impacting on the TSs of **TS-anti-6S-2**, **TS-syn-6S-2** and **TS-syn-6R-2**. Therefore, **TS-anti-6R-2** would be the preferred transition state to afford C2α-**7c** (Figure 2b).

### 3.2. Biological Evaluation of the 19-Nor Type C2 Alkoxy-Substituted D_3_ Derivatives

#### 3.2.1. Evaluation of the VDR Binding Affinity of **7a**–**c**

The VDR binding affinity of C2-alkoxy-substituted 19-nor D_3_ derivatives **7a**–**c** was evaluated by the VDR competition assay with fluorescence polarization (PolarScreen™, Invitrogen) [27], and the results are summarized in Table 2. In the case of C2α-**7a** (2α-OMe), the binding affinity (mP value) was found to be 100.8% when the value for1,25D_3_ was normalized to 100%. On the other hand, C2β-**7a** (2β-OMe) showed a lower VDR binding affinity than C2α-**7a** (47.5%). In the case of **7b**, the binding affinities of C2α-**7b** (2α-OBn) and C2b-**7b** (2β-OBn) were 2.5% and 0.4%, respectively, and almost no binding was observed in the case of C2α-**7c** (2α-*p*-NO_2_-PhO). These results suggest that a smaller alkoxy substituent at C2 with α-stereochemistry tends to show a higher VDR binding affinity [8]. From these results, it is considered that the smaller substituent with the alpha con-figuration at the C2 position would construct an appropriate interaction between the substituent and the ligand-binding domain (LBD) in the VDR, and shows a stronger VDR-binding affinity [28].

#### 3.2.2. Evaluation of the HL-60 Cell Differentiation-Inducing Activity of **7a**–**c**

It is known that 1,25D_3_ induces the differentiation of the HL-60 cells into macrophages. Thus, the differentiation-inducing activities of the new 19-nor type derivatives, **7a**–**c**, were evaluated by the NBT reduction method [21,22], and the EC_50_ values and ratios normalized to 1,25D_3_ are summarized in Table 2. Surprisingly, the differentiation activity ratio of C2α-**7a** was ca 26-fold greater than that of 1,25D_3_, even though the VDR binding affinity was similar to that of 1,25D_3_ (entry 1). The cell differentiation-inducing activities of C2β-**7a** and C2α-**7b** were similar or lower than those of 1,25D_3_ (1.41- and 0.30-fold, respectively). On the other hand, C2β-**7b** and C2α-**7c** did not show significant cell differentiation-inducing activities (ratios of 0.08- and 0.02-fold, respectively).

Thus, the 19-nor derivative with a methoxy substituent at C2α showed characteristic potent cell differentiation-inducing activity. Since the activities of the corresponding methyl- and hydroxy-substituted 19-nor D_3_ derivatives were reported to be 0.02- and 1-fold, respectively [8,9,15], the presence of a small alkoxy group appeared to have a substantial impact on the cell differentiation-inducing activity in the D_3_ derivatives. These results provide a new insight into the relationship between the structure and activity of D_3_ analogs, especially for the 19-nor type analogs, and should be helpful in the design of more potent derivatives, with better a separation of the D_3_ activities associated with binding to the VDR.

## 4. Conclusions

In summary, we have investigated the stereoselectivity in the Julia-type coupling reaction of the A ring ketones **5** and the CD ring synthon **6**. In this reaction, the steric size of the substituent at C2 of **5** affects the stereoselectivity, and the C2β product **7** is preferentially obtained when the substituent at C2 is small, while the C2α product is generated when the substituent is large. These results can be explained in terms of the stability of the transition state, which is affected by steric repulsion involving the TBS ether group on the A ring, the substituent at C2 and the CD ring moiety. Among the C2-alkoxy-substituted 19-nor type D_3_ derivatives **7a**–**c**, C2α-**7a** showed 26-fold more potent cell differentiation-inducing activity than 1,25D_3_ (**1**).

## Figures and Tables

**Table 1:**
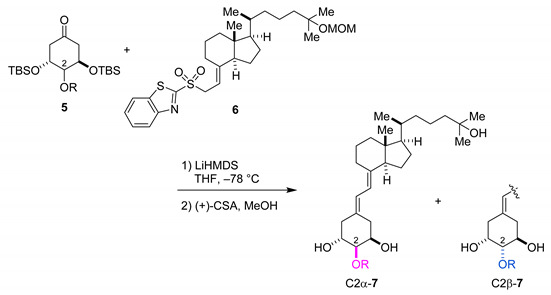
Diastereomeric ratio of coupling product **7** in Julia-type coupling of ketone **5** with sulfone **6**.

		Coupling Product 7		
Entry	Ketone 5		Yield [%] ^1^	Ratio [C2α/C2β] ^2^
1	**5a** (R = Me)	**7a**	31	1:3.5
2	**5b** (R = Bn)	**7b**	27	1:1
3	**5c** (R = *p*-NO_2_-PhO)	**7c**	62	1:0

^1^ Combined yield of isomers C2α and C2β of **7**. ^2^ Determined by ^1^H NMR.

**Table 2:** Relative VDR binding affinity and the HL-60 cell differentiation-inducing activity of C2-alkoxy substituted 19-nor type D_3_ derivatives, **7a**–**c**.

	VDR Binding	HL-60 Differentiation	
Compound	Activity Ratio ^1^	EC_50_ (M)	Activity Ratio ^1^
1,25D_3_	100.0	1.01 × 10^−8^	100
2α-OMe (C2α-**7a**)	100.8	3.80 × 10^−10^	2655
2β-OMe (C2β-**7a**)	47.5	7.13 × 10^−9^	141
2α-OBn (C2α-**7b**)	2.5	3.34 × 10^−8^	30
2β-OBn (C2β-**7b**)	0.4	1.34 × 10^−7^	8
2α-*p*-NO_2_-PhO (C2α-**7c**)	0	4.83 × 10^−7^	2

^1^ The potency of 1,25D_3_ was normalized to 100.

## Data Availability

Not applicable.

## Supplementary Materials

The following are available online at https://www.mdpi.com/article/10.3390/biom12010069/s1. Copy of ^1^H and ^13^C NMR spectra of compounds **3**, **5a**–**c** and **7a**–**c**. Figure S1: Relative VDR binding affinity of 19-norvitamin D_3_, and Figure S2: Charts of HL-60 cell differentiation activity of 19-norvitamin D_3_.
