# Supplementary material for: Synthesis of C2-Alkoxy-Substituted 19-Nor Vitamin D3 Derivatives: Stereoselectivity and Biological Activity
Source: Biomolecules. 2022 Jan 4;12(1):69. doi: 10.3390/biom12010069 (PMC8773602; doi:10.3390/biom12010069)

Supplementary material

Synthesis of C2-alkoxy-substituted 19-nor vitamin D_3 derivatives: Stereoselectivity and biological activity

Table of contents

^{1}H and ^{13}C NMR spectra of compounds **3**, **5a-c**, **7a-c** S2-S10

Figure S1: Relative VDR binding affinity of 19-norvitamin D_3 S11

Figure S2: Charts of HL-60 cell differentiation activity of 19-norvitamin D_3 S12

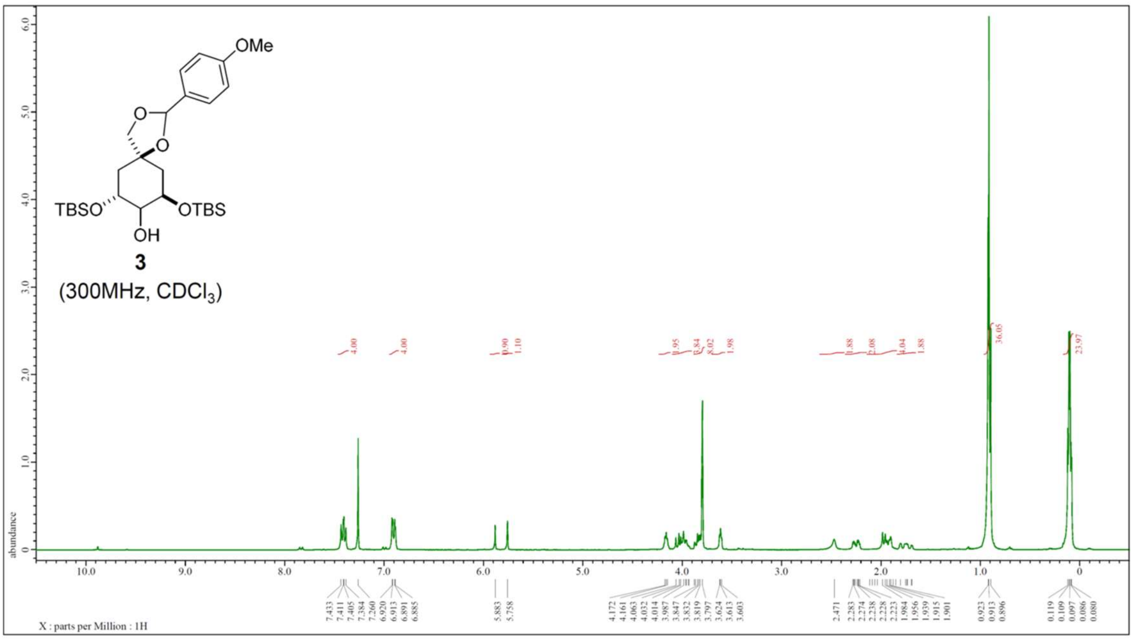
OMe
TBSO
OTBS
OH
3
(300MHz, CDCl3)
abundance
X : parts per Million : 1H

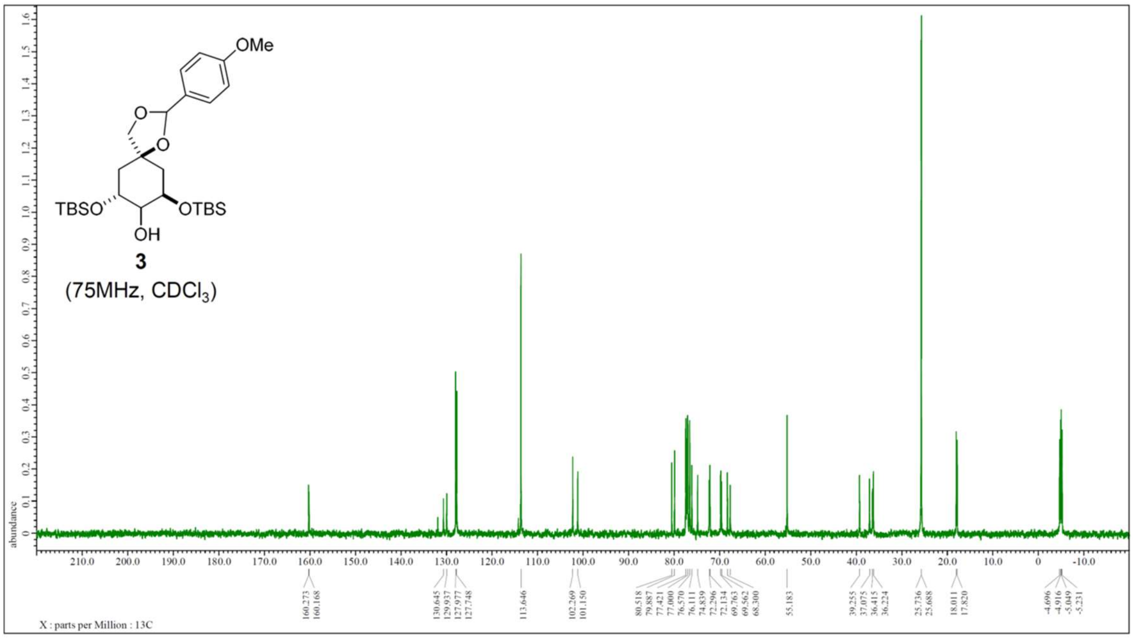
OMe
TBSO
OTBS
OH
3
(75MHz, CDCl3)
abundance
X : parts per Million : 13C

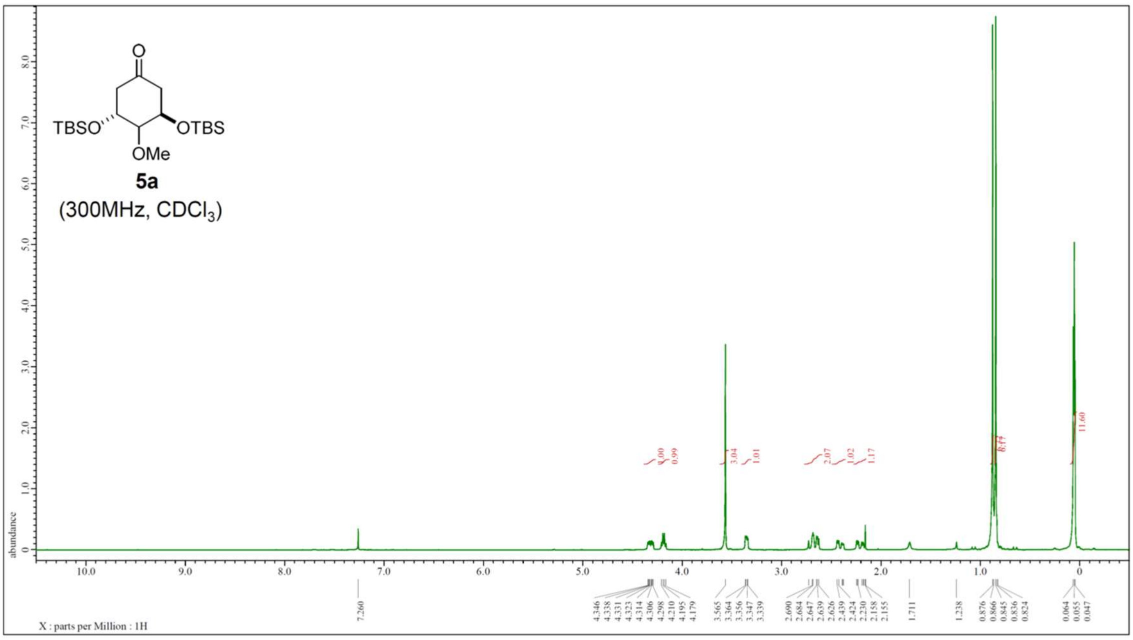
TBSO
OTBS
OMe
5a
(300MHz, CDCl3)
abundance
X : parts per Million : 1H

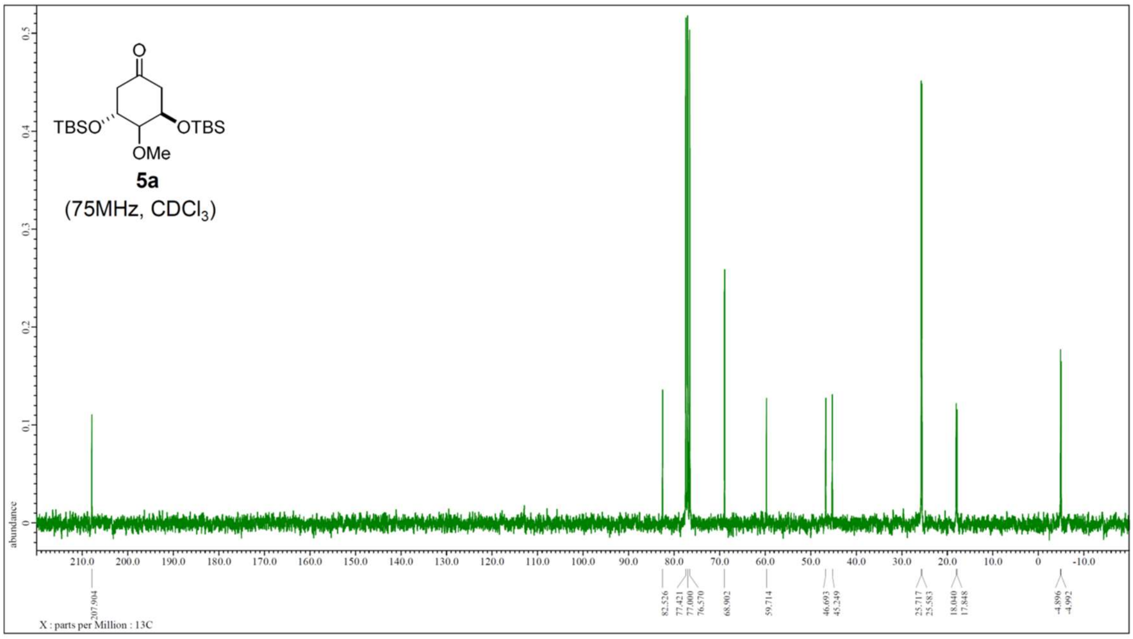
TBSO
OTBS
OMe
5a
(75MHz, CDCl3)
abundance
X : parts per Million : 13C

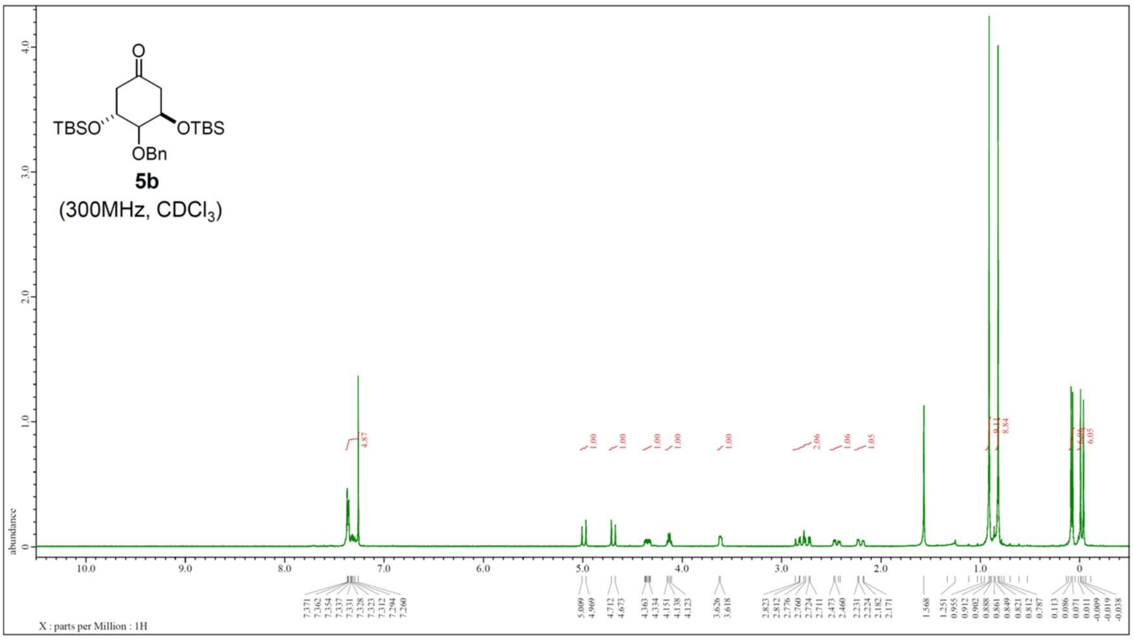
TBSO
OTBS
OBn
5b
(300MHz, CDCl3)
abundance
X : parts per Million : 1H

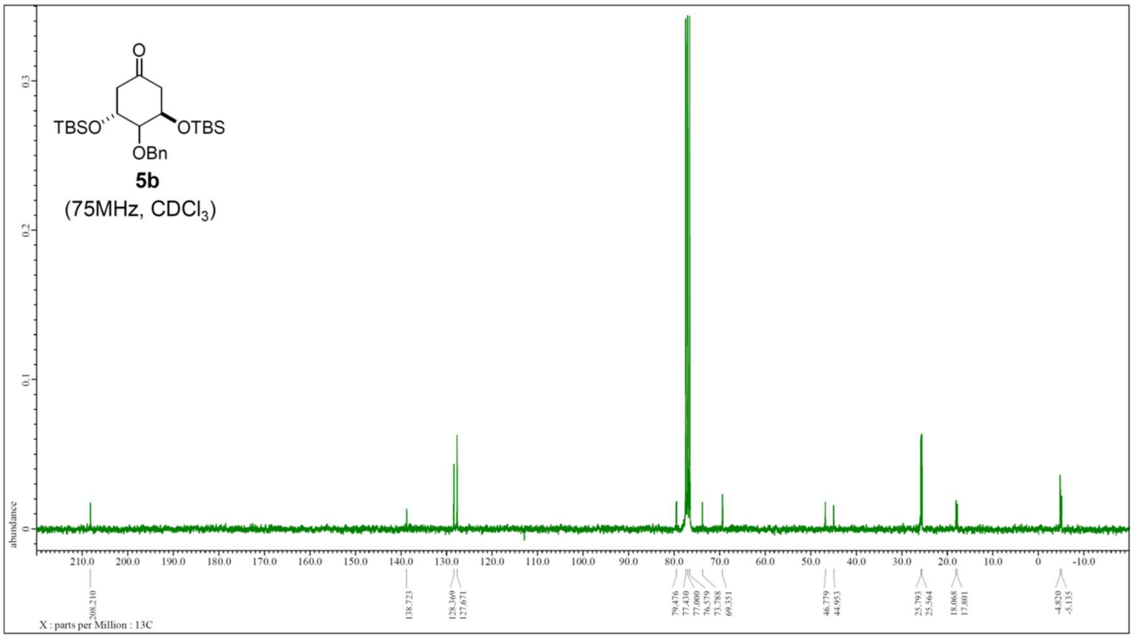
TBSO
OTBS
OBn
5b
(75MHz, CDCl3)
abundance
X : parts per Million : 13C

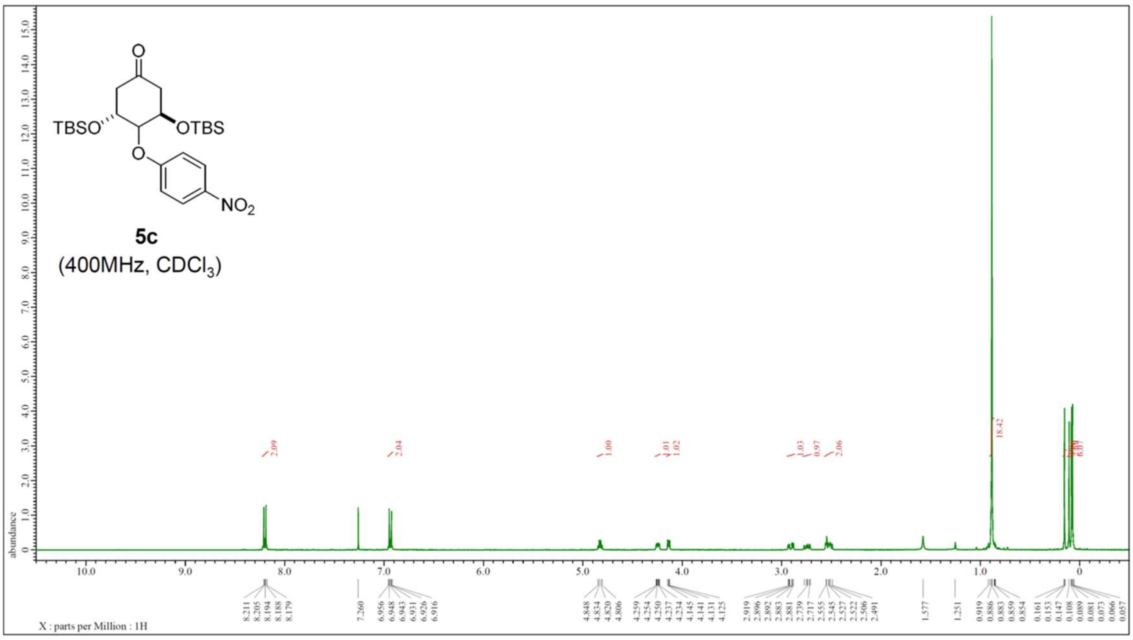
TBSO
OTBS
O
O
NO2
5c
(400MHz, CDCl3)
abundance
X : parts per Million : 1H

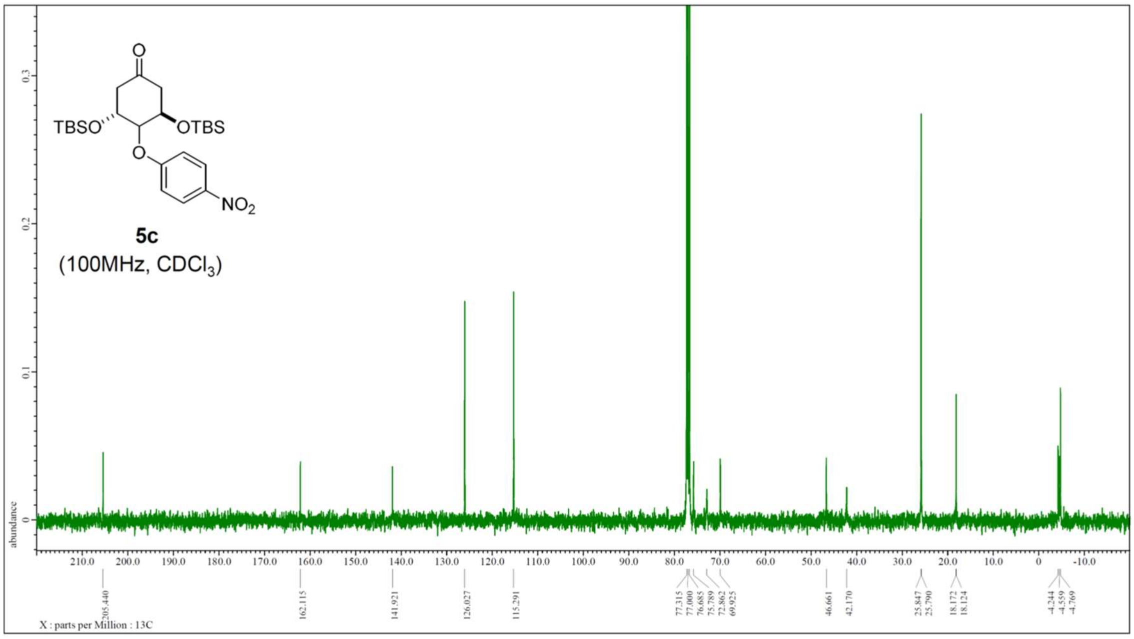
TBSO
OTBS
O
O
NO2
5c
(100MHz, CDCl3)
abundance
X : parts per Million : 13C
205.440
162.115
141.921
126.027
115.291
77.315
77.000
76.685
75.789
72.862
69.925
46.661
42.170
25.847
25.790
18.172
18.124
-4.244
-4.559
-4.769

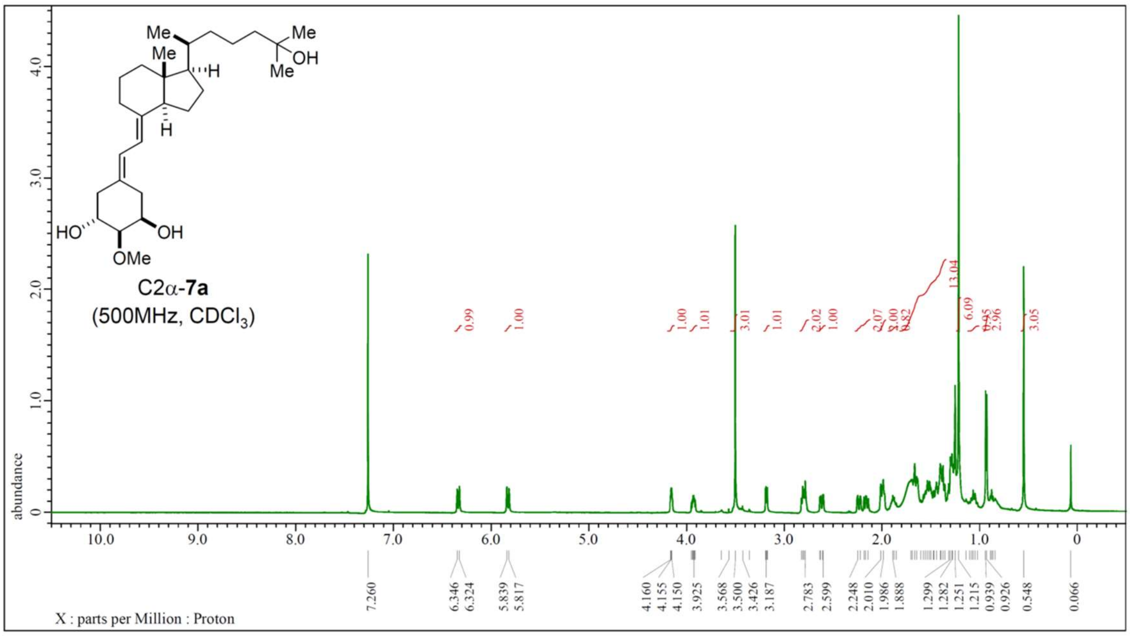
C2α-7a
(500MHz, CDCl3)
abundance
X : parts per Million : Proton

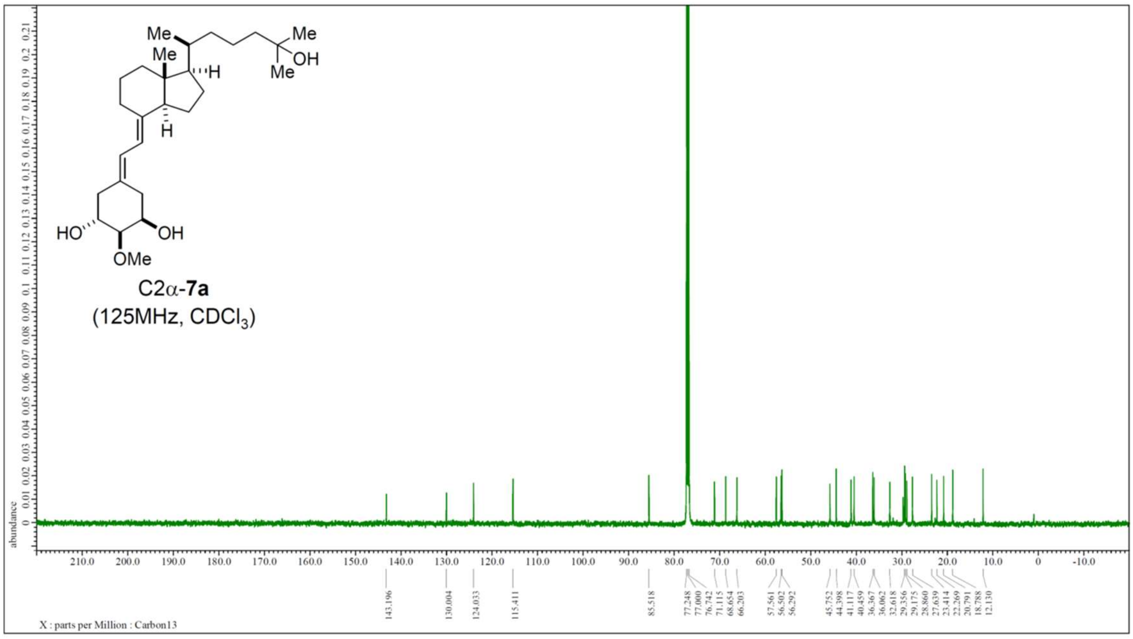
C2α-7a
(125MHz, CDCl3)
abundance
X : parts per Million : Carbon13

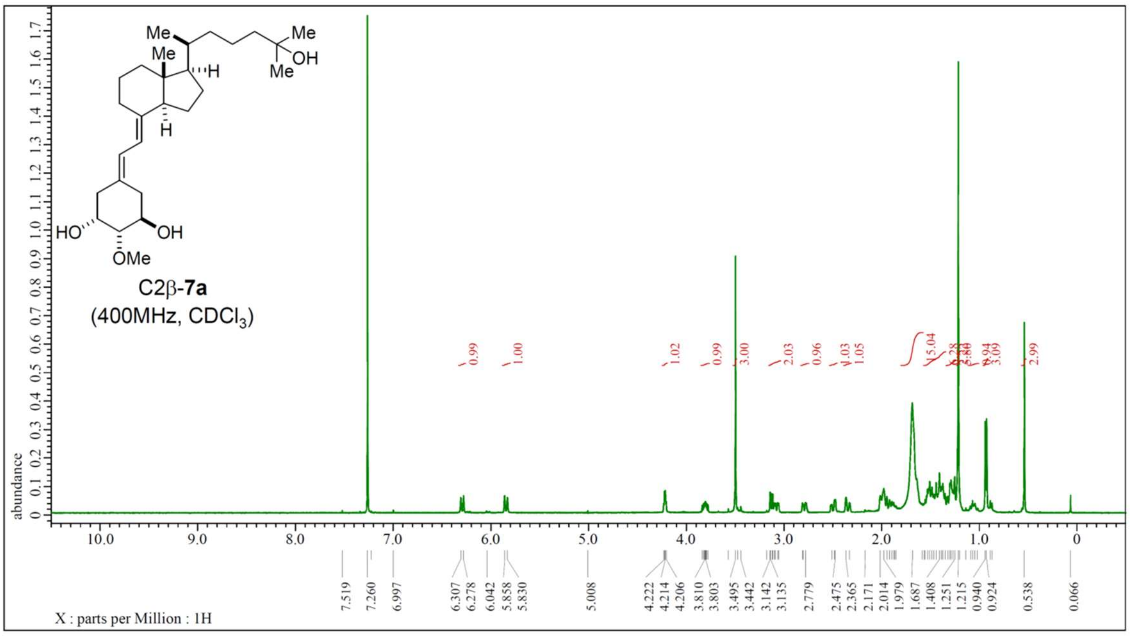
C2β-7a
(400MHz, CDCl3)
abundance
X : parts per Million : 1H

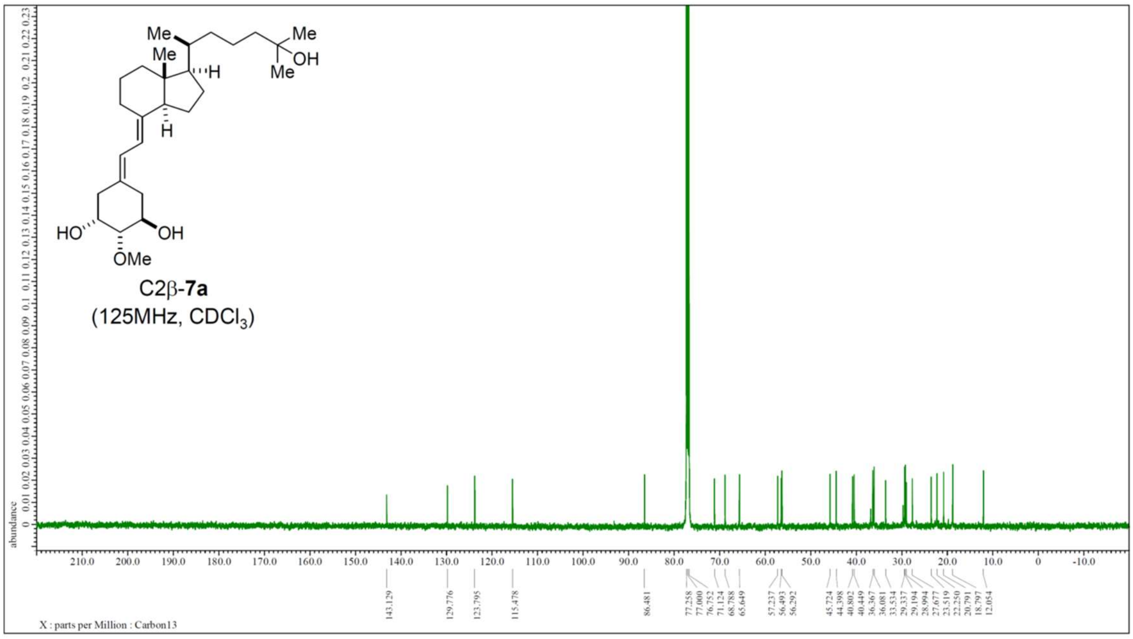
C2β-7a
(125MHz, CDCl3)
abundance
X : parts per Million : Carbon13

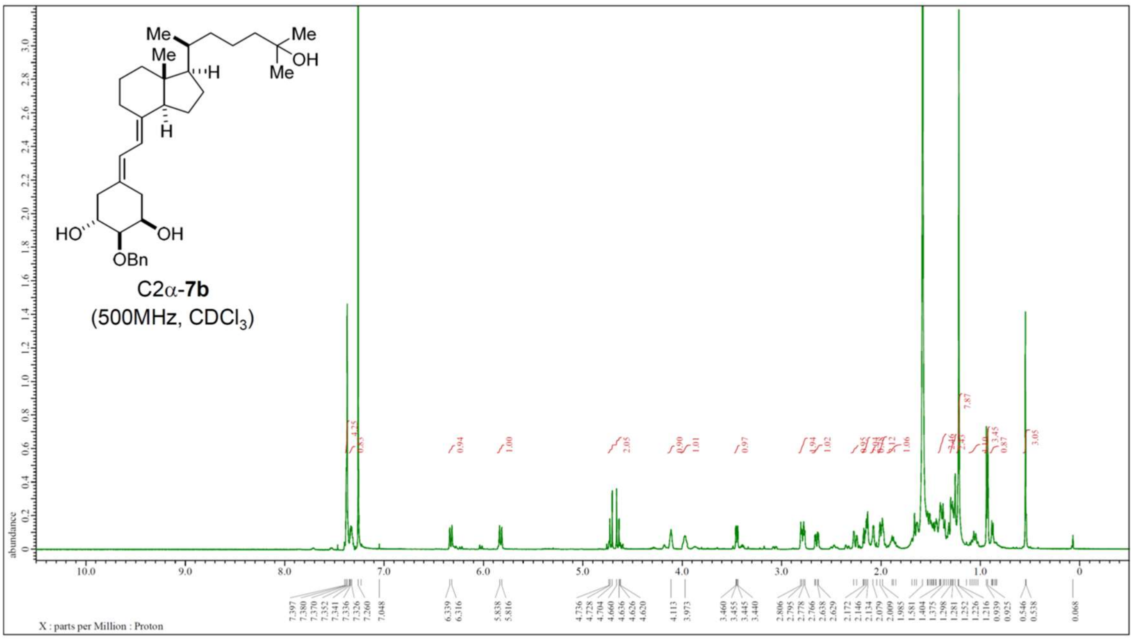
C2α-7b
(500MHz, CDCl3)
abundance
X : parts per Million : Proton

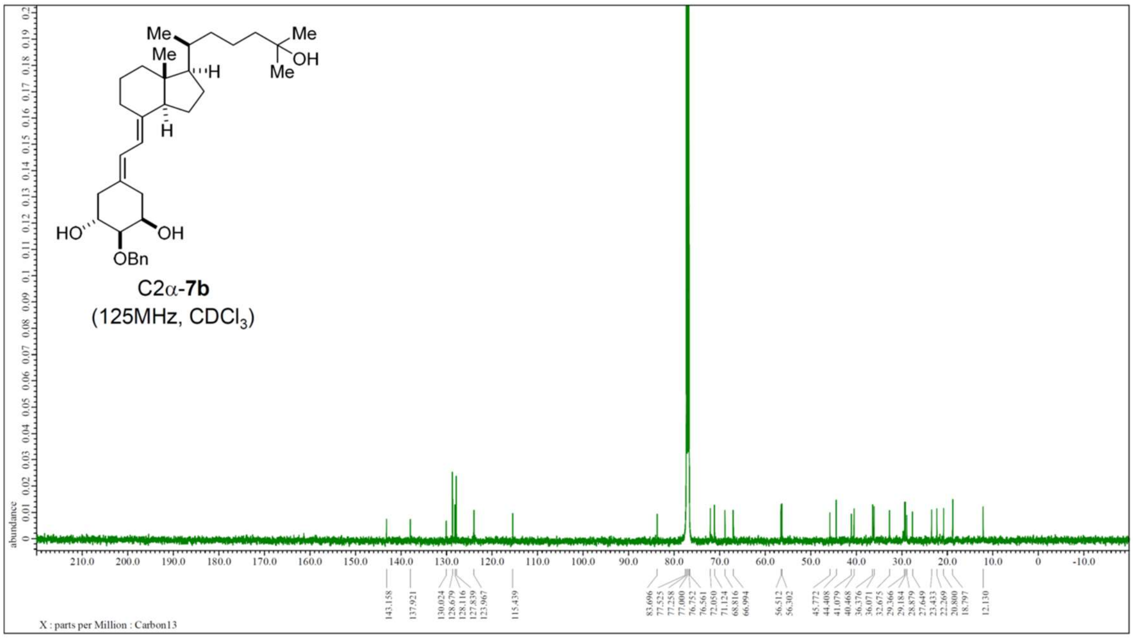
C2α-7b
(125MHz, CDCl3)
abundance
X : parts per Million : Carbon13

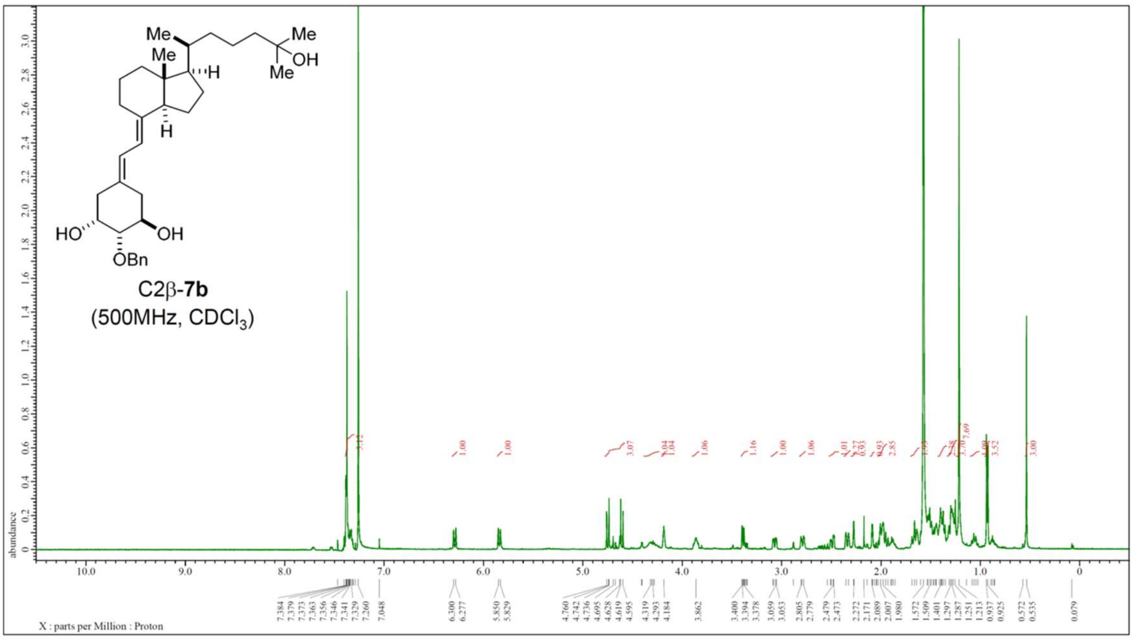

Me
Me
H
Me
OH
Me
H
HO
OH
OBn
C2β-7b
(500MHz, CDCl3)
abundance
X : parts per Million : Proton

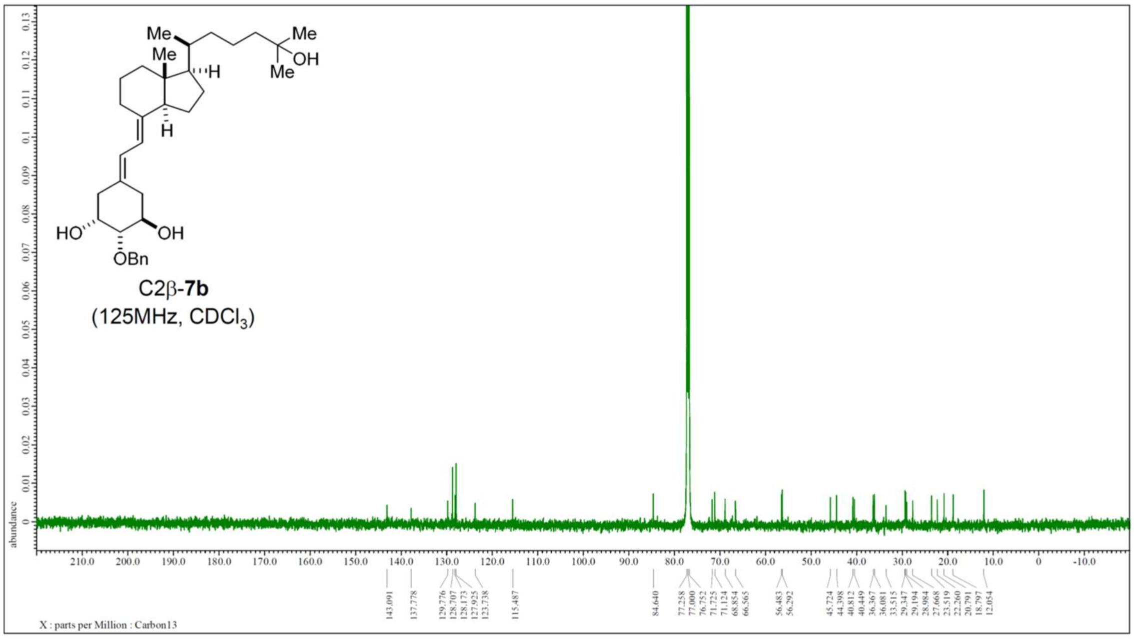

Me
Me
H
Me
OH
Me
H
HO
OH
OBn
C2β-7b
(125MHz, CDCl3)
abundance
X : parts per Million : Carbon13

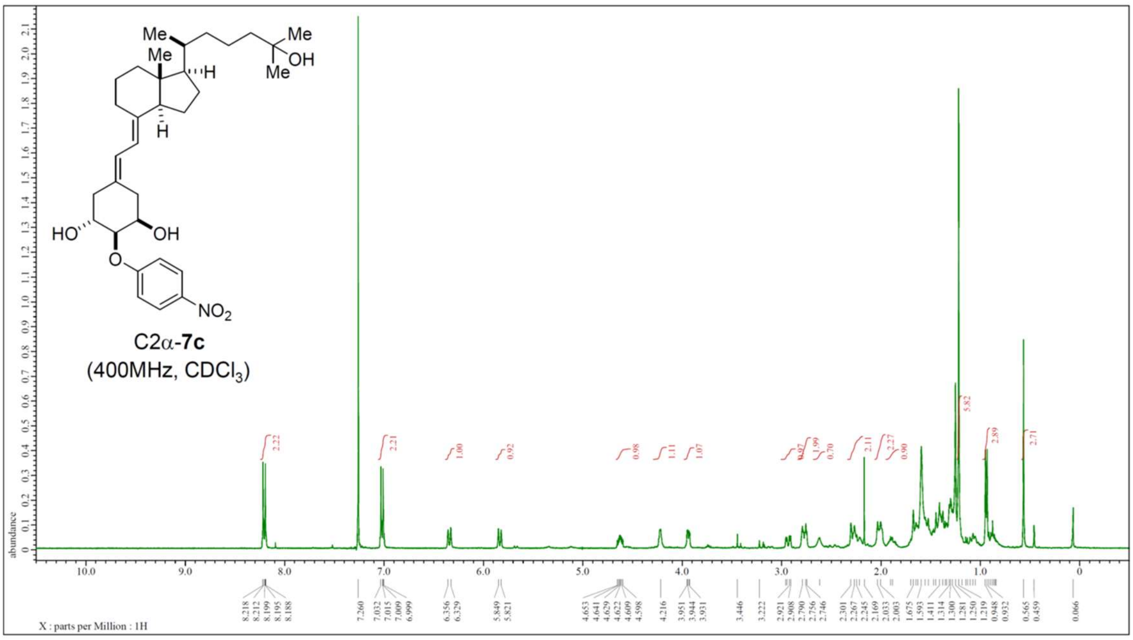
Me
Me
OH
Me
H
H
HO
OH
O
NO2
C2α-7c
(400MHz, CDCl3)
abundance
X : parts per Million : 1H

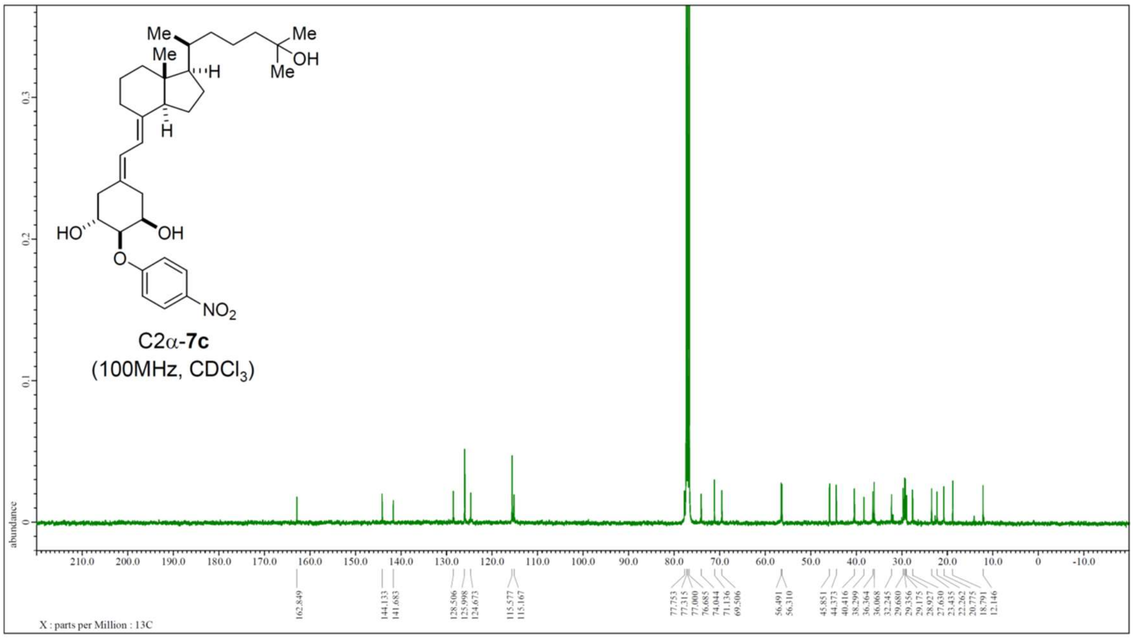
Me
Me
OH
Me
H
H
HO
OH
O
NO2
C2α-7c
(100MHz, CDCl3)
abundance
X : parts per Million : 13C

Figure S1: Relative VDR binding affinity of 19-norvitamin D3

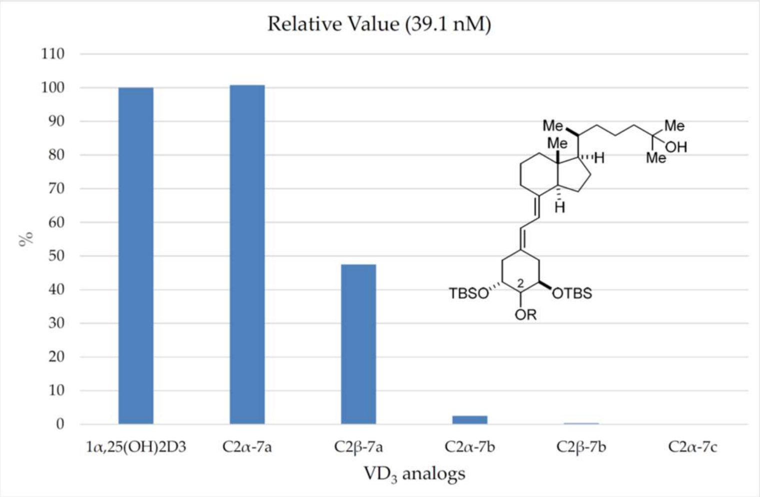

Compound	ratio
1,25VD$_3$	100
**C2α-7a** (R=Me)	100.76
**C2β-7a** (R=Me)	47.48
**C2α-7b** (R=Bn)	2.51
**C2β-7b** (R=Bn)	0.36
**C2α-7c** (R=Ph-4-NO_2)	0.00

Figure S2: Charts of HL-60 cell differentiation activity of 19-norvitamin D_3

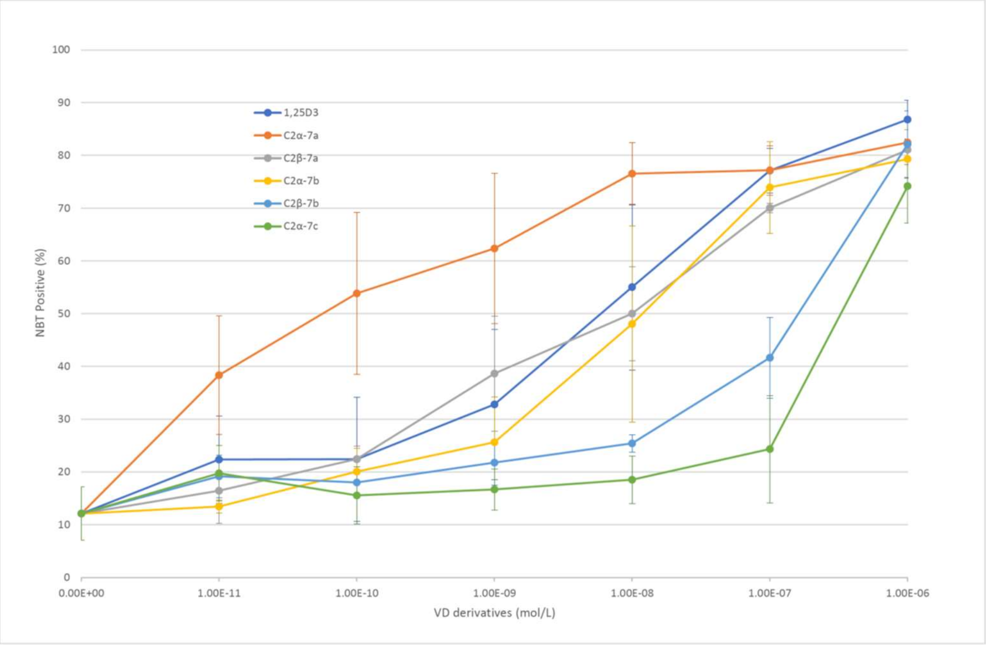

Supplement: Supplementary file 1 [file biomolecules-12-00069-s001.zip › biomolecules-1532833-supplementary.pdf]
